# New Insights into Cardiovascular Diseases Treatment Based on Molecular Targets

**DOI:** 10.3390/ijms242316735

**Published:** 2023-11-24

**Authors:** Armanda Wojtasińska, Joanna Kućmierz, Julita Tokarek, Jill Dybiec, Anna Rodzeń, Ewelina Młynarska, Jacek Rysz, Beata Franczyk

**Affiliations:** 1 Department of Nephrocardiology, Medical University of Lodz, ul. Zeromskiego 113, 90-549 Lodz, Poland; 2 Department of Nephrology, Hypertension and Family Medicine, Medical University of Lodz, ul. Zeromskiego 113, 90-549 Lodz, Poland

**Keywords:** cardiovascular diseases, dyslipidemia, PCSK9 inhibitors, ANGPTL3, bempedoic acid, siRNA, diabetes, SGLT-2 inhibitors, GLP-1 receptor agonists, VCP/p97

## Abstract

Cardiovascular diseases (CVDs) which consist of ischemic heart disease, stroke, heart failure, peripheral arterial disease, and several other cardiac and vascular conditions are one of the most common causes of death worldwide and often co-occur with diabetes mellitus and lipid disorders which worsens the prognosis and becomes a therapeutic challenge. Due to the increasing number of patients with CVDs, we need to search for new risk factors and pathophysiological changes to create new strategies for preventing, diagnosing, and treating not only CVDs but also comorbidities like diabetes mellitus and lipid disorders. As increasing amount of patients suffering from CVDs, there are many therapies which focus on new molecular targets like proprotein convertase subtilisin/kexin type 9 (PCSK9), angiopoietin-like protein 3, ATP-citrate lyase, or new technologies such as siRNA in treatment of dyslipidemia or sodium-glucose co-transporter-2 and glucagon-like peptide-1 in treatment of diabetes mellitus. Both SGLT-2 inhibitors and GLP-1 receptor agonists are used in the treatment of diabetes, however, they proved to have a beneficial effect in CVDs as well. Moreover, a significant amount of evidence has shown that exosomes seem to be associated with myocardial ischaemia and that exosome levels correlate with the severity of myocardial injury. In our work, we would like to focus on the above mechanisms. The knowledge of them allows for the appearance of new strategies of treatment among patients with CVDs.

## 1. Introduction

Cardiovascular diseases (CVDs) which consist of ischemic heart disease, stroke, heart failure, peripheral arterial disease, and several other cardiac and vascular conditions are some of the most common causes of death worldwide [1]. In 2017, CVDs caused an estimated 17.8 million deaths [2]. The most classic risk factors are high blood pressure, insulin resistance or diabetes mellitus (DM), high body mass index, air pollution, tobacco use (including smoking and passive smoking), impaired kidney function, lead exposure, alcohol use, physical inactivity, unhealthful dietary intake, and dyslipidemia which leads to atherogenesis [3]. Atherogenesis develops due to increased levels of atherogenic lipoproteins in the plasma: apoB and non-high-density lipoprotein cholesterol (non-HDL-C) [4,5].

As mentioned above, diabetes is one of the risk factors of CVDs, however, it also co-occurs with them. DM contributes to both microvascular (retinopathy, nephropathy, neuropathy) and macrovascular (ischemic heart disease, stroke) diseases and moreover–hyperglycemia may lead to atherosclerosis [6,7].

CVDs and chronic kidney disease (CKD) have similar risk factors and influence each other by worsening the status of one other. Except for these shared risk factors, CKD is an independent risk factor for CVDs [8].

We need to search for new risk factors and pathophysiological changes to create new strategies for preventing, diagnosing, and treating CVDs. In our work, we would like to focus on molecular mechanisms between CVDs and their most important risk factors: DM and dyslipidemia. The knowledge of those mechanisms allows for the appearance of new strategies of treatment among patients with CVDs.

## 2. Dyslipidemia, Its Association with Cardiovascular Disease, and New Insights in Treatment New Insights in Treatment of Dyslipidemia

Dyslipidemias are one of the most frequently diagnosed and managed medical conditions. They are characterized by an elevated plasma concentration of total cholesterol (TC), low-density lipoprotein cholesterol (LDL-C), triglycerides (TGs), and a diminished level of high-density lipoprotein cholesterol (HDL-C). The accurate values of lipid profile parameters are determined based on cardiovascular risk. The classification of the cardiovascular disease (CVD) risk group to which a patient belongs depends on systematic coronary risk evaluation (SCORE) chart results and, among other factors, the presence of comorbidities, such as diabetes mellitus (DM) and chronic kidney disease (CKD) [9].

The underlying causes of hyperlipidemia can be genetic, including familial chylomicronemia syndrome (FCS), familial dysbetalipoproteinemia (FD), familial hypertriglyceridemia (FHTG), homozygous familial hypercholesterolemia (HoFH), or autosomal recessive hypercholesterolemia (ARH). Additionally, secondary causes of dyslipidemia encompass type 2 diabetes mellitus, obesity, chronic renal failure, cholestasis, or the use of medications such as steroids, thiazide diuretics, and selected beta-adrenergic blockers [10]. Hyperlipidemia is a predominant contributor to morbidity and is strongly linked to an increased risk of atherosclerotic cardiovascular disease (ASCVD) [11,12]. Elevated levels of low-density lipoprotein (LDL) particles in the plasma, particularly those subjected to oxidation, are captured by arterial wall macrophages, leading to the formation of foam cells, which constitute the foundation of atherosclerotic plaques. Lp(a) lipoprotein particles, structurally akin to LDL, also engage with macrophages, intensifying LDL oxidation. Oxidized LDL particles initiate the inflammatory cascade, contributing to atherogenesis [13]. The rupture of resulting atherosclerotic plaques and subsequent vessel occlusion may precipitate myocardial infarction, limb ischemia, or stroke. Hypertriglyceridemia is also a significant risk factor for premature ASCVD and represents a pivotal role in atherogenic dyslipidemia and arterial inflammation [13,14].

Numerous guidelines underscore the importance of a healthy diet and prudent lifestyle practices for the primary prevention of dyslipidemia [15]. The main objective of prevention efforts is to lower LDL-C levels according to the individual’s cardiovascular disease (CVD) risk category [9]. The reduction in cholesterol levels correlates directly with a decrease in ASCVD risk, with a 20% relative risk reduction for every 1 mmol/L reduction of LDL-C [16].

Currently, the basis of dyslipidemia management are statins, ezetimibe, proprotein convertase subtilisin/kexin type 9 (PCSK9) inhibitors, and conventional lipid-lowering therapies (LLT). Despite their pivotal role in ASCVD prevention, many patients fail to attain their target lipid levels.

Due to its prevalence and the consequences of untreated, long-term dyslipidemia, this area has been the subject of extensive research, particularly focusing on pharmacological treatments and ASCVD prevention strategies. Many of these approaches hold the potential to enhance outcomes in patients with dyslipidemia, reducing the long-term consequences of the condition and the cardiovascular risk.

### 2.1. Alirocumab and Evolocumab

Alirocumab (Praluent) is a human immunoglobulin G1 monoclonal antibody [17] that acts to decrease LDL levels by inhibiting PCSK9 [18], particularly in individuals receiving statin therapy [19]. Through its interaction with hepatocyte receptors, alirocumab enhances the uptake of plasma LDL-C by liver cells [20]. In addition to lowering LDL-C, alirocumab also exhibits favorable effects on non-HDL-C, apolipoprotein B, apolipoprotein A, and lipoprotein A [17]. The study conducted by Huang AC et al. on rats demonstrated that alirocumab decreases the levels of thiobarbituric acid reactive substances (TBARS), which are oxidative stress markers while increasing the levels of glutathione peroxidase. This suggests the involvement of alirocumab in reducing oxidative stress *as* [21]. Clinical evidence indicates that alirocumab is associated with reduced cardiovascular events and mortality, particularly in patients with recent acute coronary syndrome. Notably, larger relative risk reductions were observed in patients with an estimated glomerular filtration rate (eGFR) greater than 60 mL/min/1.73 m^2^ [22].

Evolocumab (Repatha^®^, Amgen, Thousand Oaks, CA, USA) is a human immunoglobulin G2 monoclonal antibody that similarly inhibits PCSK9 and significantly lowers levels of low-density lipoprotein (LDL) cholesterol [23,24], achieving reductions of up to 60% [23]. This medication is indicated for the treatment of mixed dyslipidemia, primary hypercholesterolemia, and HoFH, particularly in patients who are ineligible for statin therapy [25]. Evolocumab has demonstrated its efficacy in reducing cardiovascular risk [23,26,27,28] and has a reduction effect on the regression of atherosclerotic plaque [27]. Concurrent use of statin and evolocumab after a non-ST-segment elevation myocardial infarction leads to stabilization and regression of atherosclerotic plaque in coronary vessels [29]. Evolocumab achieves a similar effect in patients with well-controlled LDL-cholesterol levels with statins, without inducing adverse effects [30].

### 2.2. Evinacumab

Angiopoietin-like protein 3 (ANGPTL3) plays a pivotal role in the regulation of lipid metabolism through its inhibition of plasma lipases [31,32], which are responsible for lipolysis of triglyceride-rich lipoproteins [33]. The inhibition of ANGPTL3’s action is associated with a reduction in lipid levels, consequently leading to a diminished risk of ASCVD [34]. Evinacumab, a monoclonal antibody, is directed against ANGPTL3 [35]. It has demonstrated its efficacy in reducing both LDL-C and TG levels [36,37]. Administered subcutaneously at a weekly dose of 450 mg, evinacumab reduced LDL-C levels by 56% when compared to standard treatment in patients with severe refractory hypercholesterolemia, regardless of the presence of FH [37]. Furthermore, evinacumab exhibits a synergistic lipid-lowering effect when used in conjunction with PCSK9 inhibitors and statins [38]. Ongoing research is currently investigating the potential of evinacumab in the treatment of FCS and severe hypertriglyceridemia (HTG), with initial results appearing promising [39].

### 2.3. Bempedoic Acid

A new therapeutic approach for dyslipidemia includes bempedoic acid (8-hydroxy-2,2,14,14-tetramethylpentadecanoic acid). It functions as a prodrug that intervenes in cholesterol synthesis by disrupting the action of ATP-citrate lyase (ACLY) upstream of 3-hydroxy-3-methylglutaryl CoA reductase (HMGCR) in the liver [40]. Unlike statins, bempedoic acid does not activate in skeletal muscle, potentially mitigating side effects such as rhabdomyolysis [41]. This medication is indicated for adults with heterozygous familial hypercholesterolemia (HeFH) or established ASCVD [42]. It can be administered as a monotherapy or in conjunction with ezetimibe or PCSK9 inhibitors, yielding a more significant reduction in LDL-C levels compared to the maximum dose of statins [13]. A daily dosage of 180 mg of bempedoic acid reduces LDL-C levels by up to 20% [43], and when combined with ezetimibe, it can achieve reductions of 38–50% [42,43]. Furthermore, it has been demonstrated to lower TG levels, as well as levels of glucose and high-sensitivity C-reactive protein (hs-CRP) [43,44]. It has been demonstrated that following 12 weeks of bempedoic acid usage, the level of hsCRP, an established prognostic marker for future CV events, decreased by 31.9% compared to the baseline value. Additionally, when combined with ezetimibe at a fixed dose, a 35.1% reduction in hsCRP levels was also observed after 12 weeks of therapy. These findings indicate that bempeidic acid may have anti-inflammatory effects [43]. Ongoing research is currently investigating the impact of bempedoic acid on cardiovascular morbidity and mortality [41].

### 2.4. Icosapent Ethyl 

In order to reduce the risk of cardiovascular events, such as myocardial infarction or stroke, among patients at a high risk of such events with elevated fasting triglyceride (TG) levels (>1.7 mmol/L) who are concurrently taking statins, the most recent guidelines from the National Institute for Health and Care Excellence (NICE) recommend the use of icosapent ethyl (Vazkepa) [45]. As a measure for secondary prevention, this drug is for patients with established cardiovascular disease (CVD) and LDL-C levels exceeding 1.04 mmol/L but not exceeding 2.60 mmol/L. Moreover, in the context of primary prevention, Vazkepa is recommended for individuals afflicted with DM and who possess at least one additional cardiovascular risk factor [45].

**Figure 1:**
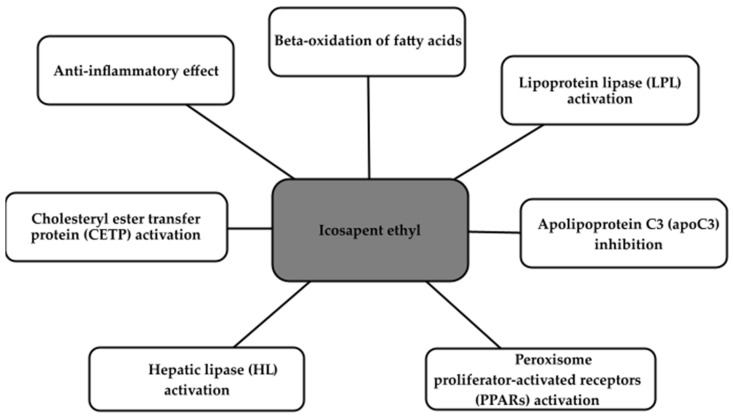
Main effects of icosapent ethyl.

The mode of action of Vazkepa encompasses various critical facets, including the augmentation of fatty acid beta-oxidation, resulting in the reduction in very low-density lipoprotein (VLDL) synthesis. Furthermore, it enhances lipoprotein lipase (LPL) activity, inhibits apolipoprotein C3 (apoC3), activates peroxisome proliferator-activated receptors (PPARs), hepatic lipase (HL), and cholesterol ester transfer protein (CETP). Beyond its influence on hepatic lipogenesis, Vazkepa also exerts anti-inflammatory and antioxidant effects. These collective actions culminate in the potential to reduce the deposition of fatty deposits in the blood vessels, thereby preventing their blockage [46]. The main effects of Vazkepa are shown in the Figure 1.

### 2.5. SiRNA Therapy 

The current LDL-lowering effect of statins turns out to be insufficient. Inhibition of PCSK9 has been proven to enhance this effect by intracellular degradation of LDL cholesterol receptors and thus reduce their recycling and expression on the hepatocyte membrane [47]. However, it is still too weak for many patients, and they also complain about many side effects [48]. A promising new therapeutic approach is based on small interfering RNAs [49]. siRNA is a small double-stranded RNA that works by dividing into single strands and binding to their distinct messenger RNA (mRNA) target sequences. As a result, the target mRNA breaks and is degraded, which additionally stops translation and induces gene suppression by short RNA strands. This is also called RNA interference (RNAi) [50,51,52]. As a result, it causes increased recycling of hepatocytes and membrane expression of LDL receptors, and a reduced level of LDL-C [53].

Therefore, siRNA technology is associated with an improvement in the lipid profile, thereby contributing to a reduction in the incidence of serious adverse cardiac events and hospitalizations due to heart failure and strokes compared to placebo [54]. It is worth mentioning a few other positive features of this technology: potential inhibitory effect on the expression of an unlimited number of genes, high selectivity, and reversibility (siRNA does not lead to permanent modification of the genome), and may become cheaper to produce [55].

Four agents are currently approved by the FDA: patisiran, givosiran, lumasiran, and inclisiran. They are particularly recommended for the treatment of adult patients with hereditary transthyretin amyloidosis (hATTR), acute hepatic porphyria (AHP), primary hyperoxaluria type 1 (PH1), and for lowering LDL-C in patients with heterozygous familial hypercholesterolaemia (HeFH) or clinical atherosclerotic cardiovascular disease (ASCVD) [56].

Patients with familial hypercholesterolemia (FH) have high cholesterol levels in their blood from birth, increasing their risk of atherosclerosis in the heart, brain, and peripheral arteries, and a significantly higher risk of cardiovascular events and death [57]. The Dutch Lipid Clinic Diagnostic Criteria (DLCN) ≥ 6 points (diagnosis of definite or probable FH) and DLCN < 3 points (diagnosis of unlikely FH) are effective in predicting these events [58]. Inclisiran reduces LDL-C levels by over 50% with one dose every 6 months [59].

The results obtained so far from the ORION (A Randomized Trial Assessing the Effects of Inclisiran on Clinical Outcomes Among People with Cardiovascular Disease) study confirmed that inclisiran effectively reduced LDL-C in patients despite maximally tolerated statin therapy (± ezetimibe) in various clinical conditions [60]. The ORION-3 trial reported that long-term exposure to inclisiran (up to 5 years) as well as switching from evolocumab to inclisiran are both safe and effective in achieving and maintaining LDL-C reduction [61].

In general, inclisiran has been well-tolerated with mild and transient injection-site reactions being the primary side effect. Its impact on reducing cardiovascular events is still under investigation, with some positive results in composite major adverse cardiovascular events (MACE). However, more extensive trials like ORION-4 and VICTORION-2 PREVENT will provide a clearer picture of its long-term safety and efficacy [62,63].

### 2.6. Other Therapy Solutions 

Aptamers (oligonucleotides or peptides that bind specifically to a specific molecule) represent a promising solution for the purpose of measuring LDL concentration in blood. Aptamers show affinity and specificity similar to monoclonal antibodies. Moreover, they are non-immunogenic and have high tissue penetration, similar to small molecules [64]. The aptamers and their corresponding antisense strands demonstrate sufficient affinity and specificity for LDL particles to be used in a simple, clinically relevant diagnostic test to measure LDL-P in a cheap and rapid manner [65]. They may be an attractive alternative to antibodies for analytical applications (they are well known for troponin I, troponin T, myoglobin, and C-reactive protein), but so far they are not widely used in practice in diagnostics and medical research [66]. Current research on aptamers in the diagnosis of cardiovascular diseases focuses on their potential use in point-of-care imaging tests and a wider range of laboratory tests [67].

Another interesting drug is pelacarsen—an antisense oligonucleotide directed to the liver, which strongly reduces the level of lipoprotein(a) [Lp(a)]. Lp[a] is strongly associated with atherosclerotic disease and aortic stenosis. Lp(a) forms by bonding between apolipoprotein(a) (apo[a]) and apo B100 [68]. In the HORIZON trial, in which a monthly dose of 80 mg was administered to healthy Japanese subjects, a strong reduction in plasma Lp(a) concentration was observed [69].

Another new drug that lowers Lp(a) levels is muvalaplin. It is an oral drug that inhibits Lp(a) formation by blocking the apo(a)-apo B100 interaction, avoiding interaction with the homologous protein, plasminogen. When administered daily to patients for 14 days, it reduced Lp(a) levels by up to 65% [70].

## 3. The Association between Cardiovascular Disease and Diabetes

Cardiovascular disease (CVD) remains the leading cause of death in patients with type 2 diabetes, while diabetes is one of the fastest growing diseases worldwide, which might significantly affect the overall quality of life [7,71]. Cardiovascular morbidity and mortality in patients with diabetes are too high to be fully explained by traditional cardiovascular risk factors (like smoking, hypertension, dyslipidemia) [72,73].

**Figure 2:**
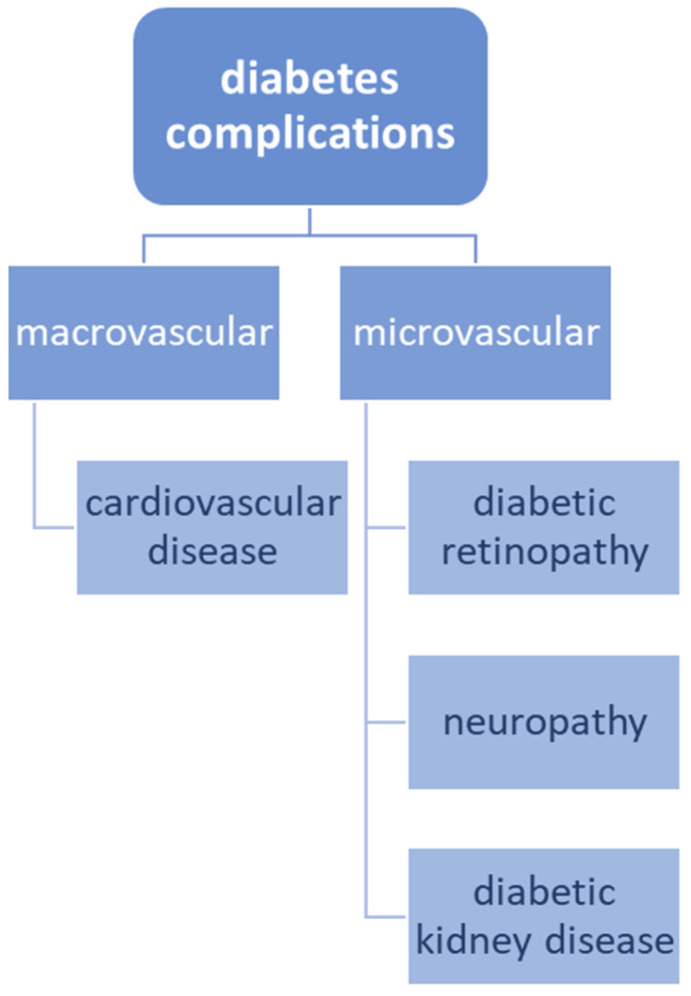
Main division of diabetes complications based on the size of the affected vessels.

Diabetes complications are typically divided into macrovascular (cardiovascular disease) and microvascular (diabetic retinopathy, neuropathy, and diabetic kidney disease) and this classification is presented in Figure 2 [7]. The evidence has shown that microvascular disease in diabetes can predict atherosclerotic CVD, including stroke and myocardial infarction [74,75]. Indeed, mechanistic studies suggest that microvascular and macrovascular complications of diabetes may share common molecular pathways, such as increased oxidative stress, interrupted protein kinase C signaling, accumulation of advanced glycation end products (AGEs) in vessel walls, and endothelial dysfunction, which might all lead to vascular inflammation, vasoconstriction, thrombosis and atherosclerosis [76,77].

Advanced glycation end products are modifications of proteins, lipids, or nucleic acids that undergo non-enzymatic glycation and oxidation following exposure to aldoses [78]. AGEs can fluoresce, generate reactive oxygen species (ROS) and bind specific cell surface receptors [76]. Interaction of AGEs with their major cellular receptor—receptor for advanced glycation end products (RAGE), activates multiple signaling pathways, such as tumor growth factor beta (TGF-β), the c-Jun N-terminal kinase (JNK), mitogen-activated protein kinase/extracellular signal-regulated kinases (MAPK/ERK), and nuclear factor kappa-light-chain-enhancer of activated B cells (NF-κB), leading to increased oxidative stress and inflammation [78]. The hyperglycemic environment is a vital factor in the process of formation of AGEs, which further contribute to the pathophysiology of diabetic vascular disease during aging. AGEs could accumulate in blood vessel walls, resulting in damage to cell structure and function [76,79].

Although intensive treatment of diabetes reduces the risk of major macrovascular and microvascular events by ≥10%, clinical risk factors and glycemic control alone do not predict the development of vascular complications; many genetic studies have shown that diabetes and its complications have a strong genetic component as well [7,76].

## 4. The Role of SGLT-2 Inhibitors and GLP-1 Receptor Agonists in Diabetes and Cardiovascular Disease

### 4.1. Sodium-Glucose Co-Transporter-2 (SGLT-2) Inhibitors

Sodium-glucose co-transporter-2 (SGLT-2) inhibitors were initially created to lower glucose levels in patients with diabetes, however, it has been found that they may provide additional benefits such as decreasing blood pressure [80]. Moreover, clinical trial data demonstrate that these medications can protect against cardiovascular disease, particularly by lowering the risk of hospitalization due to heart failure in patients with both reduced and preserved ejection fraction [80,81]. These positive effects were observed in patients with or even without diabetes diagnosis [82].

The main representatives of oral SGLT-2 inhibitors include empagliflozin and dapagliflozin [83]. Their mechanism of action relies on lowering glucose reabsorption in the proximal tubule by 50–60%, which results in increased excretion of glucose in the urine and reduced plasma glucose level [84]. Nonetheless, SGLT-2 inhibitors have the potential to reduce the risk of CVD through multiple diverse and complex processes. These drugs can reduce adipose tissue-mediated inflammation and inhibit the production of pro-inflammatory cytokines, leading to beneficial anti-inflammatory effects. Furthermore, SGLT-s inhibitors are involved in lowering oxidative stress, inhibiting advanced glycation end products (AGEs) signaling and decreasing serum uric acid levels. Due to increased early natriuresis and hence, decreased plasma volume, these molecules contribute to lower blood pressure and improved vascular function [82].

### 4.2. Glucagon-like Peptide-1 (GLP-1) Receptor Agonists

Glucagon-like peptide-1 (GLP-1) receptor agonists present a hypoglycemic activity due to the incretin effect, which causes increased meal-induced insulin secretion in comparison to parental glucose intake [85]. These drugs mimic the action of Glucagon-like peptide-1 (GLP-1)—an incretin hormone released from pancreatic beta cells as a response to a meal [86]. Activation of GLP-1 receptor not only improves glucose tolerance but could also protect pancreatic islet ß cells and stimulate their proliferation, present anti-inflammatory and cardioprotective function, control lipid metabolism, and support the growth of nerves [87]. Additional biological actions of GLP-1 agonists include appetite suppression and delayed stomach emptying, which may result in loss of weight [88].

All of the abovementioned effects are very beneficial for patients with type 2 diabetes, but they might be favorable for the general population as well. Liraglutide, semaglutide, and albiglutide are the representatives of GLP-1 agonists which have presented the ability to reduce the risk of major adverse cardiac events (MACE) in many studies [88,89,90,91]. Decreased cardiovascular risk significantly contributes improved glucose and lipid metabolic profile, cardioprotective function, lower body weight, and decreased blood pressure [92].

## 5. The Emerging Significance of VCP/p97 in Cardiovascular Ailments: Fresh Insights and Therapeutic Prospects

### 5.1. VCP/p97′s Involvement in Cardiovascular Conditions 

VCP/p97 is the ATPase valosin-containing protein. Disrupted protein equilibrium is a hallmark of diverse cardiovascular afflictions [93], encompassing myocardial infarctions, heart failures, and diabetic cardiomyopathies. VCP/p97 is believed to play a pivotal role in maintaining protein balance within the cardiovascular system.

For instance, transgenic mice expressing VCP/p97 K524A exhibit cardiomyopathy marked by the accumulation of ubiquitinated proteins [94]. Moreover, VCP/p97 assumes a critical role in bolstering cardiomyocyte survival, which, in turn, underpins the preservation of mitochondrial function [95].

### 5.2. VCP/p97 and Ischemia/Reperfusion-Induced Damage 

**Figure 3:**
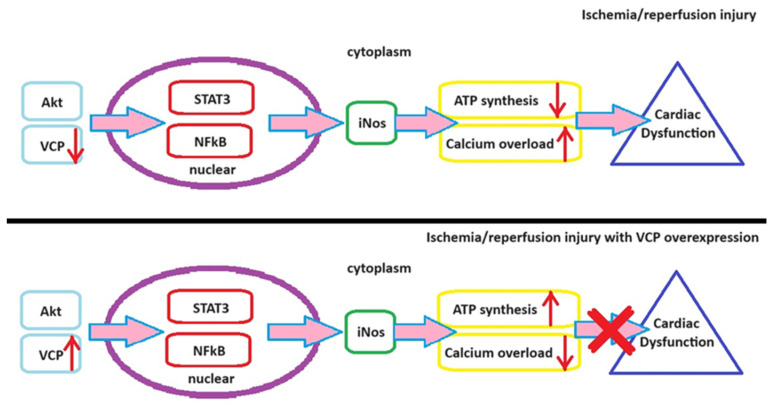
VCP’s Influence on Cardiac Ischemia/Reperfusion-Related Injuries. NF-κB—Nuclear factor kappa B. STAT3—signal transducer and activator of transcription 3.

Ischemia-reperfusion denotes a pathological state characterized by an initial restriction of blood supply to a portion of the myocardium followed by the restoration of blood flow during the reperfusion phase [96]. Consequently, ischemia and reperfusion collectively induce damage to myocardial tissue [97,98]. Employing ischemic preconditioning for ischemic myocardium effectively mitigates the harm incurred during ischemia/reperfusion events [99]. Endogenous nitric oxide (NO) synthesized in the myocardium regulates myocardial contraction and vascular vasodilation, thereby reducing the size of myocardial infarctions and enhancing endothelial function [100]. Inducible NO synthase (iNOS) is primarily responsible for NO production [52], conferring a significant role upon iNOS in ischemic preconditioning [101]. VCP/p97 plays a protective role in ischemia/reperfusion injuries, as evidenced by a 50% reduction in myocardial infarction size in VCP/p97 transgenic mice compared to their wild-type counterparts following ischemia/reperfusion events [102]. Additionally, overexpression of VCP/p97 in cardiomyocytes diminishes celastrol-induced myocardial apoptosis [103]. Further mechanistic investigations reveal that VCP/p97 enhances NO production through iNOS, as depicted in Figure 3.

VCP/p97 stimulates iNOS expression in a concentration-dependent manner, with NF-kB playing a pivotal role in this process, as indicated in Figure 2. The proteolytic effects of VCP overexpression can be nullified by the addition of the NF-kB inhibitor, SN50 [103].

Current research posits that VCP/p97 could emerge as a promising therapeutic target for ischemia-reperfusion injuries and pressure overload-induced cardiac hypertrophy, though there are dissenting perspectives that need further exploration. Extensive investigation is warranted to ascertain whether VCP/p97 possesses broader myocardial protective properties. Moreover, elucidating VCP/p97’s role in safeguarding against non-ischemic heart conditions, including diabetic cardiomyopathies and idiopathic dilated cardiomyopathies, is imperative. Furthermore, numerous cardiovascular ailments stemming from pressure overload, such as pathological cardiac hypertrophy, result in disturbances in protein homeostasis [104]. Hence, it is imperative to probe into the mechanisms through which VCP/p97 upholds protein equilibrium within the myocardium.

## 6. Fresh Insights and Novel Perspectives on Exosomes in Cardiovascular Disorders

### 6.1. Exosomes as Diagnostic Tools in Cardiovascular Ailments

Given that cardiovascular diseases (CVDs) can induce pathological alterations in cardiac tissue, Sluijter et al. [105] have proposed that exosomes derived from various sources might serve as valuable biomarkers for diagnosing diverse CVDs. Exosomes, which carry a cargo determined by the cell types and conditions of their origin (e.g., miRNAs, proteins, lncRNAs), are produced abundantly by a variety of cells and actively participate in a wide array of cardiovascular processes, both normal and pathological [106].

There is strong evidence indicating a link between exosomes and heart muscle ischemia. Exosomes, which are small particles released by cells, seem to be connected to the extent of heart muscle damage. In situations where there is low oxygen (hypoxia) or reduced blood flow (ischemia) in the heart, cells release a large number of exosomes carrying specific genetic material called miRNAs into the bloodstream. This process results in higher levels of exosomes in patients with cardiovascular disease (CVD) and those who have experienced a heart attack (acute myocardial infarction, AMI) [105]. For example, one study found significantly heightened levels of circulating exosomal miR-133a, primarily originating from infarcted and peri-infarcted myocardium, in acute coronary syndrome patients. Furthermore, serum miR-133a levels surged within two hours of chest pain onset, preceding the elevation of creatine kinase and troponin levels [107,108,109].

Hence, both the quantity and composition of exosomes are regarded as early and disease-specific indicators for CVDs [110]. The analysis of exosomal contents is critical for clinicians to swiftly diagnose, identify, and manage diseases while improving prognostic outcomes. Beyond their capacity to mirror physiological and pathological changes within cardiac tissue [111,112], exosomes possess the ability to safeguard their molecular contents (e.g., miRNAs, proteins, lncRNAs) from RNases, enhancing the feasibility and accuracy of diagnostics. For example, numerous circulating miRNAs, including miR92a/b, miR1, miR499, miR133, and miR122, exhibit overexpression in CVD patients [113,114]. However, miRNAs or lncRNAs on their own are not stable in circulation and are susceptible to enzymatic degradation. Therefore, comprehensive genomics or proteomics analyses of exosome profiles could offer greater accuracy. Moreover, exosomes are easily obtainable from a variety of bodily fluids, including blood, urine, plasma, and semen, making their use in current clinical practice feasible [115]. In summary, exosomes offer distinct advantages as diagnostic tools for CVDs.

### 6.2. Exosomes as Therapeutics in Cardiovascular Ailments

Apart from their ability to diagnose diseases, exosomes can also aid in repairing cardiac tissues by influencing cellular processes, both normal and disease-related. Current medical approaches often struggle to repair the loss of heart muscle cells following a heart attack. Scientists are now concentrating on creating therapies centered around cells to encourage the growth and reactivation of these heart muscle cells, known as cardiomyocytes [116].

Nonetheless, limitations such as low transplant cell survival rates, limited capacity for differentiation into functional cardiomyocytes, immune rejection, and other factors hinder the clinical application of stem cell therapy [117]. Mounting evidence suggests that exosomes derived from stem cells can play a pivotal role in cardiac repair. Studies have demonstrated that stem cells confer cardioprotection through autocrine and paracrine mechanisms. Given the diverse contents of exosomes, including enriched miRNAs, growth factors, lipids, and proteins, they can facilitate on-site cardiomyocyte proliferation and activation, leading to the regeneration of infarcted tissue [118,119].

Furthermore, exosomes exhibit the ability to evade phagocytosis and lysosomal engulfment while eliciting minimal immune responses, thus enhancing their therapeutic efficacy [120,121]. Exosomes sourced from various cell types have shown comparable levels of cardioprotection to their parent cells in preclinical experiments [122,123]. Exosomes employed in CVD treatment can be categorized based on their cellular origins, including cardiac resident cells and stem cells.

## 7. Conclusions

Nowadays, CVDs are one of the common causes of death worldwide so we need to treat them effectively to prevent their complications. There are multiple researches which describe new targets of treatment.

The need to search for new strategies in dyslipidemia’s treatment appeared because many patients fail to attain their target lipid levels. Alirocumab and evolocumab are human immunoglobulins monoclonal antibodies that similarly inhibit PCSK9 and significantly lower levels of low-density lipoprotein (LDL) cholesterol even up to 60%. Bempedoic acid can be administered as a monotherapy or in conjunction with ezetimibe or PCSK9 inhibitors, yielding a more significant reduction in LDL-C levels compared to the maximum dose of statins. A promising new therapeutic approach is based on small interfering RNAs, mainly inclisiran. New molecules for the treatment of dyslipidemia will certainly be created, but it is worth mentioning that due to their cost, these are not yet common therapies available to patients.

In diabetes mellitus treatment, there are medicines which focus on sodium-glucose co-transporter-2 and glucagon-like peptide-1. SGLT-s inhibitors are involved in lowering oxidative stress, inhibiting advanced glycation end products (AGEs) signaling, and decreasing serum uric acid levels. Due to increased early natriuresis and hence, decreased plasma volume, these molecules contribute to lower blood pressure and improved vascular function. GLP-1 receptor agonists decrease cardiovascular risk significantly by contributing to improved glucose and lipid metabolic profile, cardioprotective function, lower body weight, and decreased blood pressure.

There are other molecular mechanisms important in the development of CVDs such as exosomes which are considered early and disease-specific biomarkers for CVDs however they need to be further researched.

## Data Availability

Not applicable.
